# Microengineered neuronal networks: enhancing brain-machine interfaces

**DOI:** 10.1097/MS9.0000000000002130

**Published:** 2024-05-06

**Authors:** Burhan Kantawala, Ali Emir Hamitoglu, Lea Nohra, Hassan Abdullahi Yusuf, Kirumira Jonathan Isaac, Sanobar Shariff, Abubakar Nazir, Kevin Soju, Konstantin Yenkoyan, Magda Wojtara, Olivier Uwishema

**Affiliations:** a Oli Health Magazine Organization, Research and Education, Kigali, Rwanda; b Neuroscience Laboratory, Cobrain Centre, Yerevan State Medical University named after Mkhitar Heratsi, Yerevan, Armenia; c Faculty of Medicine, Namik Kemal University, Tekirdag, Turkey; d Faculty of Medical Science, Lebanese University, Beirut, Lebanon; e College of Health science, Faculty of Clinical Sciences Bayero University Kano, Nigeria; f Faculty of Clinical Medicine and Dentistry, Kampala International University, Uganda; g Department of Medicine, King Edward Medical University, Pakistan; h Faculty of Medicine, Christian Medical College, Ludhiana, India; i Department of Biochemistry, Yerevan State Medical University named after Mkhitar Heratsi, Yerevan, Armenia

**Keywords:** brain–machine interface (BMI), devices, disorders, ethics, microengineered neuronal networks (MNNs), neurology, therapeutics

## Abstract

The brain–machine interface (BMI), a crucial conduit between the human brain and computers, holds transformative potential for various applications in neuroscience. This manuscript explores the role of micro-engineered neuronal networks (MNNs) in advancing BMI technologies and their therapeutic applications. As the interdisciplinary collaboration intensifies, the need for innovative and user-friendly BMI technologies becomes paramount. A comprehensive literature review sourced from reputable databases (PubMed Central, Medline, EBSCOhost, and Google Scholar) aided in the foundation of the manuscript, emphasizing the pivotal role of MNNs. This study aims to synthesize and analyze the diverse facets of MNNs in the context of BMI technologies, contributing insights into neural processes, technological advancements, therapeutic potentials, and ethical considerations surrounding BMIs. MNNs, exemplified by dual-mode neural microelectrodes, offer a controlled platform for understanding complex neural processes. Through case studies, we showcase the pivotal role of MNNs in BMI innovation, addressing challenges, and paving the way for therapeutic applications. The integration of MNNs with BMI technologies marks a revolutionary stride in neuroscience, refining brain–computer interactions and offering therapeutic avenues for neurological disorders. Challenges, ethical considerations, and future trends in BMI research necessitate a balanced approach, leveraging interdisciplinary collaboration to ensure responsible and ethical advancements. Embracing the potential of MNNs is paramount for the betterment of individuals with neurological conditions and the broader community.

## Introduction

Highlights

- The brain–machine interface (BMI), a crucial conduit between the human brain and computers, holds transformative potential for various applications in neuroscience. This manuscript explores the role of micro-engineered neuronal networks in advancing BMI technologies and their therapeutic applications.
- The advancement of research in BMIs has been facilitated by the utilization of microengineered neural networks, marking a substantial contribution to the field. Case studies within BMI research underscore their pivotal role. In a notable study, individuals with disabilities achieved precise control over robotic arms through the utilization of microfabricated electrode arrays.
- An integral aspect of this exploration involves the study of synapses and neurotransmitter activity, essential for grasping signal processing at the cellular level. Such investigations contribute valuable insights into the mechanisms underlying memory, learning, and other cognitive processes. Another paramount concern in neuroscience involves understanding how the spatial organization of a neural network influences its activity.

The brain–machine interface (BMI), also referred to as the brain–computer interface (BCI), serves as a vital conduit facilitating orchestrated interactions between the human brain and computers, enabling purposeful device utilization in response to cognitive processes and signals originating from the brain^1^. These neurological signals are recorded and transmitted to execute predefined functions, embodying a direct neuronal interface for communication between the brain and external devices^2^. Fundamentally, BMI establishes connections between the brain and computers through scalp, subdural, or subcortical electrodes, while the emergence of micro-physiological systems (MPS) offers novel approaches to in vitro modeling of normal and diseased physiology^3–5^.

Applications of BCIs in neuroscience are diverse, ranging from medical condition monitoring to seizure identification and mitigation, facilitating interaction for individuals with severe neurological deficits, and real-time correlation of observable behavior with neural signals^2^. The transformative potential of BMI technologies in addressing various conditions in individuals with severe neuronal debilitation underscores the need for enhanced innovation and understanding, fostering interdisciplinary collaboration across neuroscientists, computer scientists, engineers, psychologists, and rehabilitation specialists^6^. Moreover, BMI’s application extends to individuals with motor but not cognitive brain damage, spinal cord injuries, multiple sclerosis, amyotrophic lateral sclerosis, and other conditions impairing muscle movement, emphasizing the importance of improving their quality of life through external devices^7^.

The purpose of micro-engineered neuronal networks (MNN) in advancing BMI lies in their pivotal role as a link connecting the human brain to machines, facilitating communication and comprehension between individuals and the nervous system^8^. Deciphering the intricate physiological and pathological mechanisms underlying neuronal network interconnections necessitates simultaneous detection of electrophysiological and chemical signals in the brain, highlighting the criticality of neuronal interfaces in wireless BMI^9^.

The aim of this study is to synthesize and analyze MNNs in the context of BMI technologies, with the overarching goal of enhancing our understanding of BMI mechanisms and their potential therapeutic applications. By bridging the gap between neuroscience and engineering, this research contributes to the advancement of BMI technologies and their translation into clinical practice.

## MNN: construction and methodological insights

Functional activities in the human body are orchestrated by neurons and their interconnected networks, which engage in parallel processing. The emergence of MNN stems from the endeavor to replicate these intricate neural circuits artificially, particularly with advancements in 3D technologies facilitating enhanced communication between the brain and external devices^5,10^.

### Fabrication methods

The design of micro-physiological models is tailored based on desired functions, such as neural MPS customized for studying neural tube defects^11^. Various nanofabrication and microfabrication methods are employed for this purpose. Topographical nanoscale and microscale components, along with three-dimensional microenvironments, are integrated into brain-on-chips using microsystems technology^5^. These components aid in monitoring the multimodal cell culture parameters and the input of advanced chip-based nano-bioanalytical and micro-bioanalytical methods. Etching is a technique in micro-electromechanical system fabrication, which transfers structures into bulk materials like silicon and glass or thin films, followed by the removal of materials using selective etchants and chemicals^5^. Soft lithography is another method which involves transferring features from a template to an elastomeric material, enabling the creation of small structures quickly, easily, and affordably^5^. The development of organs-on-chips and multiorgan MPS involves incorporating patterns mimicking neuronal tissue structures, utilizing soft materials as scaffolds, implementing nano-topography, and ensuring long-term stability in functionality^12^. Recent advancements in MPS have been facilitated by the discovery of biocompatible materials with minimal adverse effects, such as polydimethylsiloxane (PDMS), and the application of methods like PDMS-free microchips designed through computer-aided processes and flow profile characterization^4^.

### Biomimetic approach

The biomimetic approach to neuronal replication focuses on optimizing structures by replicating the topology of biological neuronal networks in both 2D and 3D biomimetic neuronal structures^13^. Research efforts have showcased the fabrication of biomimetic neuron structures, demonstrating a viable method to replicate topological features of biological neuronal networks extensively^13^. The practical implications of biomimetic and 3D neural networks (NNs) are observed in applications such as cochlear implants for hearing restoration, where electrical stimuli are delivered inside the cochlea, highlighting the advancements achieved through the integration of biomimetic principles into neural engineering^14^.

## Applications of MNNs

### Purpose in advancing BMI technologies

MNNs play a crucial role in advancing BMI technologies by providing a sophisticated platform for understanding neural circuitry and its interface with external devices. These networks serve as indispensable tools in wireless BMI systems, facilitating precise decoding and interpretation of neural signals for seamless communication between the brain and external devices^15^.

### Specific examples and evidence

Case studies within BMI research illustrate the indispensable role of MNNs. For instance, in a groundbreaking study, individuals with disabilities achieved precise control over robotic arms using microfabricated electrode arrays that recorded and decoded brain signals from cultured neurons, demonstrating the potential for movement restoration^15^. Another notable case study involved the establishment of a closed-loop system where MNNs modeled neural diseases and responded to drug interventions, contributing to the development of targeted treatments for conditions like epilepsy and Parkinson’s disease^16^.

### Adequacy and coverage of case studies

The presented case studies showcase the versatility and applicability of MNNs in diverse BMI research domains. They encompass precise control of robotic arms, modeling neural diseases, and improving the functionality and durability of BMIs in communication devices and neuroprosthesis (NPs)^17^. These case studies collectively highlight the broad impact of MNNs on advancing BMI technologies and enhancing the quality of life for individuals with neurological conditions or disabilities^16^.

### Impact on functionality and quality of life

MNNs significantly enhance the performance and durability of BMI systems, leading to improved functionality in communication devices and NPs. By enabling precise control over external devices through decoded neural signals, these networks enhance the autonomy and quality of life for individuals with neurological conditions or disabilities^16^. Moreover, their potential for targeted drug interventions in neurological diseases underscores their impact on improving treatment outcomes and enhancing overall functionality and quality of life.

### Investigating neural connectivity and signal processing

In addition to their applications in BMI research, MNNs contribute to the investigation of neural connectivity and signal processing. These networks provide valuable insights into the communication dynamics among diverse brain regions during cognitive processes such as problem-solving or sensory perception^18^. Moreover, in vitro studies with microengineered networks serve as indispensable tools for probing the relationship between the structure and function of NNs, shedding light on fundamental mechanisms underlying memory, learning, and other cognitive processes^19^.

MPS offer several advantages, including 3-D structures mirroring human organs, regulated cell-cell interactions, and the capability for in situ monitoring of disease progression and drug responses^20^. These systems find applications in modeling human embryogenesis, neuroectoderm regionalization, and disease pathophysiology, providing valuable insights into disease initiation and progression^21,22^.

## Advancing our understanding of BMI technologies

### How they help us understand neural processes

MNNs furnish meticulously controlled and reproducible platforms for delving into the intricacies of the brain, thereby advancing our comprehension of neurological processes^23^. Cultivated on microfabricated substrates, these networks offer precise monitoring of brain activity and connections. Utilizing microelectrode arrays, researchers record and process signals, decoding neuronal connections and plasticity. A comprehensive understanding of these fundamental principles holds the potential to enhance insights into neural diseases, unlock advancements in BMIs, and revolutionize the field of machine learning^24^. Furthermore, these networks play a pivotal role in the development of neuro-prosthetics, BMIs, and disease modeling. By bridging the gap between in vivo complexity and in vitro control, micro-engineered systems offer indispensable insights into neural processes, crucial for advancing neuroscience and medical research^23,24^.

### The role of these networks in refining BCIs

BCIs allow the CNS to acquire new skills in which brain signals take the place of the spinal motor neurons that produce natural muscle-based skills^25,26^. MNNs can be directly integrated with neurons, offering a more intimate and precise interface compared to electrodes. This allows for recording and stimulation of individual neurons, leading to richer and more nuanced data capture^26^. Unlike electrodes, which can be invasive and cause scarring, MNNs are designed to be minimally disruptive to the brain tissue. This minimizes the risk of inflammation and long-term damage, paving the way for safer and more sustainable BMI use. MNNs can potentially eliminate the need for cumbersome wires connecting the brain to external devices. This opens up possibilities for completely wireless BMIs, granting users greater freedom and mobility^27^.

### Evidence for MNNs’ indispensability in wireless BMI

Proof-of-concept studies: Researchers have successfully implanted MNNs in animal models and demonstrated their ability to record and modulate neuronal activity. For example, MNNs could control the movement of robotic limbs in people with high accuracy^28^. Noninvasive BMIs have been used as neurorehabilitation tools primarily in clinical studies focused on stroke victims. The main assumption motivating these studies has been that practice with a BMI that mimics movements of a paralyzed limb could facilitate brain plasticity and contribute to some level of motor recovery. For example, stroke patients can learn to operate an MEG-based BMI by modulating their μ rhythm recorded in the hemisphere ipsilateral to the lesion. In this study, the BMI opened and closed an orthosis that was attached to the paralyzed hand. This learning did not cause noticeable clinical improvements. However, long-term BMI training combined with physical therapy resulted in clear motor recovery^29,30^. Patients report tangible benefits in independence, daily activities, and overall well-being. Advantages over current technologies: MNNs offer several key advantages over existing electrode-based BMIs. Their smaller size and biocompatibility reduce tissue damage and inflammatory responses^31^. Additionally, their potential for wireless communication eliminates the risk of infection and device failure associated with implanted wires.

### Bridging gaps in our knowledge of BMI technologies

BMIs hold immense promise for revolutionizing healthcare, rehabilitation, and even human augmentation. These technologies directly connect the brain to external devices, enabling communication and control in ways previously unimaginable^32^. While preliminary studies show promising results, the long-term safety and efficacy of implanted BMI devices remain unclear. Issues like tissue response, biocompatibility, and potential neural disruption require further investigation^32^.

We need to consider accessibility and inclusivity to ensure that these technologies benefit all individuals, regardless of background or ability. Stability of BMI over the long run is still difficult. The significance of creating biocompatible materials to reduce immune reactions and guarantee the longevity of implanted devices is highlighted by recent findings by Smith *et al*.^33^, 2019. While MNNs hold immense promise for advancing BMIs, it is crucial to consider alternative perspectives and technologies like Optogenetics, BCIs.

## Therapeutic potential of MNNs

The therapeutic potential of MNNs presents a promising avenue for addressing the challenges posed by neurodegenerative diseases such as stroke, traumatic brain injury, and neurodegenerative disorders. Recent studies have demonstrated significant advancements in this field, showcasing specific findings on the efficacy of MNNs in clinical applications.

### Specific findings

In a randomized trial conducted by Ramos-Murguialday *et al*., chronic stroke patients with severe hand paresis showed significant improvement in upper-limb motor assessment scores, electromyography (EMG), and functional magnetic resonance imaging (fMRI) after intervention with BMIs. These interfaces, which included voluntary desynchronization of ipsi-lesional EEG-sensorimotor rhythms triggering paretic upper-limb movements via robotic orthoses, demonstrated promising outcomes for motor function restoration^34^. Additionally, trials conducted by Prinsloo *et al*.^35^ indicated that BCIs were effective in relieving chemotherapy-induced peripheral neuropathy in cancer survivors by altering the brain’s perception of pain, offering potential long-term pain relief.

### Clinical applications

Micro-tissue engineered neural networks (micro-TENNs) represent a strategic approach aimed at simultaneously replacing lost neurons and restoring their long-distance axonal connections, particularly in the context of neurodegenerative diseases. These networks hold promise for addressing the profound challenges associated with axonal pathway loss and neuronal degeneration in conditions such as stroke and neurodegenerative disorders, offering potential therapeutic interventions to improve motor function and alleviate neuropathic pain^36,37^.

### Ethical considerations

The development and clinical deployment of BCIs and NPs raise ethical concerns regarding safety, efficacy, and patient autonomy. Ethical frameworks must address potential conflicts between treatment objectives and research pursuits, ensuring patient safety while advancing therapeutic technologies. Additionally, considerations regarding the commercialization and military applications of BCIs and NPs underscore the need for comprehensive ethical guidelines to mitigate potential risks and safeguard patient well-being^37^. Further ethical considerations will be addressed later in the manuscript (Table 1).

**Table 1:** Ethical considerations in the development of therapeutic BMIs.

Ethical considerations	Details
Treatment vs. research conflict	• Clinical application of BCIs and NPs raises concerns about treatment-research conflicts. • Balancing research objectives and patient care is an ethical priority
Inconsistency in safety and effectiveness	• BCIs and NPs vary in safety and effectiveness, posing risks to patients. • Ethical practices should evolve with technical advancements to ensure device safety in clinical use
Commercial applications and BCI overdose	• BCIs in commercial applications may risk BCI overdose, akin to addiction concerns. • Preventing misuse and overreliance on BCIs outside medical contexts. • Responsible usage of BCIs to address ethical concerns
Military applications and threats	• Military applications of BCIs and NPs raise ethical concerns, including potential threats. • Ethical practices are essential to regulate their responsible and ethical deployment in military contexts
Simultaneous development of ethical practices	• Ensuring safe development and use of devices like BCIs, with awareness of risks and threats and implementation of appropriate safety measures

Micro-Engineered Neuronal Networks: Enhancing Brain–Machine Interfaces.

## Case studies in BMI innovation

A multitude of studies have explored the evolution of BMI innovation, employing diverse technological methodologies that have significantly contributed to the field. This review emphasizes pivotal studies that showcase the broad applications of MNNs.

### Diverse representation of case studies

The selected case studies offer a diverse representation of BMI innovation, encompassing motor control, pain management, neuro-amplification, and cognitive enhancement applications. Notably, Hochberg *et al*.^38^’s study describes a BMI empowering individuals with spinal cord injuries to control a prosthetic arm in real-time using brain-implanted electrodes, exemplifying advancements in motor function restoration. Deo *et al*.^39^’s research explores the use of NN decoders for achieving high-quality bimanual control of two cursors, highlighting the potential for multieffector decoding and enhancing motor function rehabilitation.

### Representative applications

In recent advancements, Magnetoencephalography (MEG) BMI demonstrates success in alleviating intermittent pain resulting from brachial plexus root avulsion, showcasing potential applications in pain management and neural rehabilitation^40^. Additionally, the development of a BMI-based exoskeleton, utilizing EMG signals to estimate joint angles and restore functionality to paralyzed arms and hands, illustrates practical rehabilitation applications^41^. The Neuralink implant introduced by the Neuralink company aims to amplify neural activity and treat diseases originating from central or peripheral neural origins, highlighting the potential for neuro-amplification and therapeutic interventions^42^.

### Cognitive enhancement applications

**Figure 1:**
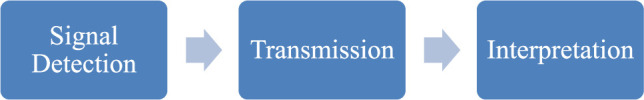
Pathway of signal communication.

Beyond motor functions, BMI shows promise in enhancing cognitive function, as demonstrated by Lee *et al*.^43^’s study on EEG-based BCI Cognitive Training (CT) program. This innovative program showcases the potential to enhance cognition in senior Chinese and English speakers, suggesting applications in cognitive rehabilitation and enhancement (Fig. 1).

These selected case studies collectively illustrate the diverse and promising avenues BMI research is exploring, ranging from motor function restoration to pain management, neuro-amplification, and cognitive enhancement. Each study represents a unique advancement in the field, highlighting the versatility and potential of MNNs in addressing a wide range of neurological challenges.

## Ethical and regulatory considerations

**Figure 2:**
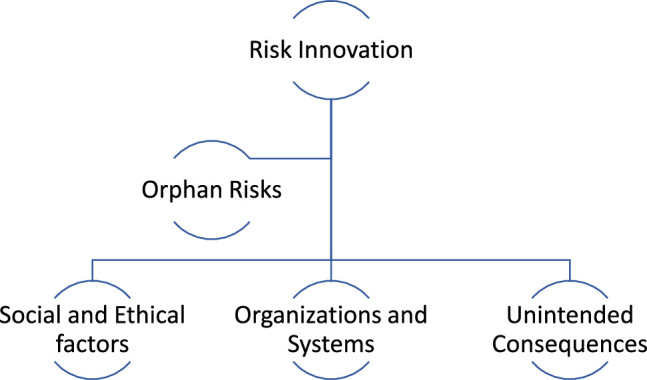
Three major domains which fall under risk innovation.

Contemporary BMIs offer transformative possibilities for treating neurological diseases and enhancing user performance. However, the rapid progression of technological advancements raises concerns that these innovations may outpace our collective understanding of how to ensure the ethical and responsible development and utilization of BMIs. The overarching concept of ‘risk innovation’ encompasses various factors that could jeopardize a company’s ability to retain qualified workers^44^. Within the realm of risk innovation, multiple risks spanning three distinct domains have been identified, as illustrated in the chart^45^ (Fig. 2).

**Figure 3:**
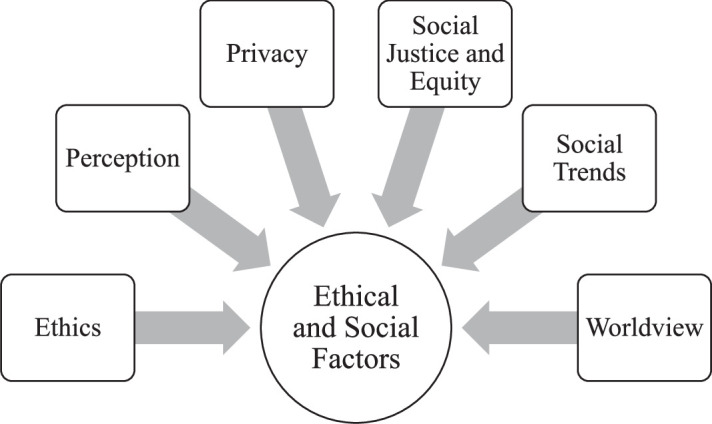
Various parameters which concerns ethical and social factors.

The innovation of BMIs brings forth a myriad of ethical dilemmas, particularly in the realms of social and ethical considerations. Firstly, there is the risk of business practices crossing the boundary from ethical to unethical conduct, emphasizing the critical need for adherence to clear guidelines and ethical standards. Secondly, on a social level, there is a concern that people’s privacy may be compromised through the use or misuse of their data, necessitating vigilant safeguards to protect individuals’ confidential information. Thirdly, accessibility issues may arise, leading to technological advancements not being universally available across all segments of society. This potential lack of accessibility can contribute to disparities and inequities by either marginalizing or favoring specific groups within society. Addressing these ethical challenges is crucial for the responsible development and deployment of such technologies (Fig. 3).

### Patient and public participation in research

A fundamental aspect of ensuring ethical development and utilization of BMIs is the active involvement of patients and the public in research processes. Patient-centered care advocacy emphasizes the importance of incorporating patient perspectives, values, and preferences into the research and development of medical technologies^46^. Recent sources highlight the significance of patient and public participation in shaping ethical considerations and regulatory frameworks surrounding BMIs^47^. By actively engaging patients and the public in research endeavors, stakeholders can better understand the needs and concerns of those directly impacted by BMI technologies, ultimately fostering more ethical and patient-centered approaches to development and deployment.

### Recent findings on patient-centered care advocacy

Recent studies emphasize the pivotal role of patient-centered care advocacy in shaping ethical and regulatory considerations in BMI research and development. For instance, Manukian *et al*.^48^ utilized machine learning algorithms to provide accurate physics simulations and subject-specific parameterization, highlighting the importance of incorporating patient-specific data and feedback into BMI design and testing processes. By integrating patient perspectives and experiences, researchers can develop more personalized and effective BMI technologies that better meet the needs and preferences of individual users.

The research led by Musk *et al*., with a predominant focus on technological advancements in BMI technology, presents a challenge when applying a risk innovation approach. The inherent subjectivity arises from the limited consideration of how these advancements might be fully integrated into future products and services. Applying a risk innovation approach to the described BMI technology, as outlined by Musk^49^, suggests numerous risks between the current state of the technology and its successful development and use.

Many of these risks revolve around how businesses navigate critical ethical and social issues associated with this technology. For instance, the potential exacerbation of the wealth gap, challenges to social norms, concerns related to autonomy, privacy implications, and the establishment of trust within the community toward developers are all significant factors that could impact the successful integration and acceptance of BMI technology. These challenges underscore the complexity and multidimensional nature of the risks that need to be carefully considered and managed throughout the development and deployment of such advanced technologies.

## Future directions, limitations, and challenges

BMIs are expected to make significant strides in the years to come due to several new developments and opportunities^46^. The incorporation of machine learning algorithms and artificial intelligence (AI) into BMI systems is one of the major trends as these technologies have the potential to improve BMI performance and flexibility, making them more user-friendly and sensitive to their needs^46^. Despite significant advancements, challenges persist in translating MNNs into practical therapeutic applications. BMIs face challenges rooted in psycho-physiological factors, including psychological traits, resting state physiological parameters, and individual differences in brain complexity. Neuro-anatomical correlations, technological limitations, and BCI illiteracy further can complicate the development of efficient systems. Technological challenges involve modalities, neuro-imaging techniques, and signal processing, requiring adaptive methods for robust classifier design^50^. Overcoming these hurdles requires addressing technical limitations such as electrode quality and surgical implantation techniques. Improved signal capture and implantation procedures are crucial for advancing the clinical feasibility of MNNs, especially in broader functional restoration goals such as communication and speech enhancement^15^ (Table 2).

**Table 2:** Challenges in the development of therapeutic BMIs.

Challenges	Details
Limited neurogenesis	• Micro-tissue engineered neural networks (micro-TENNs) aim to address the limited neurogenesis in the central nervous system. • They seek to replace lost neurons and restore axonal connections, mitigating severe and often permanent consequences for neurological function
Ethical concerns in BMI development	• Conflicts between treatment and research interests • Inconsistent safety and effectiveness • Risks of BCI overdose in commercial applications • Ethical challenges from military applications
Technical hurdles in BMI technology	• Understanding neural encoding of intentions and actions • Developing electrodes for high-quality signal capture • Refining surgical techniques for safe implantation
Scope expansion challenges	• Addressing broader aspects of human functional restoration and enhancement • Impairments especially in communication and speech • Meeting additional challenges within this scope

### Limitations and gaps

One of the primary limitations in BMI research is the long-term stability and compatibility of NNs with host tissue. While recent advancements have shown promise, ensuring the sustained functionality of these interfaces over extended periods remains a challenge. Additionally, there is a gap in understanding the long-term implications of implantable BMIs, particularly regarding patient privacy and data security. This lack of clarity poses ethical dilemmas and necessitates further research into the societal and ethical implications of BMI deployment.

Another limitation lies in the noninvasive approaches, such as electroencephalography (EEG)-based systems, which face technical obstacles in achieving robust and reliable performance. Despite their potential to enhance patient acceptance and overall experience, these systems require advancements to ensure long-term stability and compatibility with host tissue.

### Approaches to address limitations

To address the limitations in BMI research, interdisciplinary collaborations are essential. By bringing together neuroscientists, engineers, clinicians, ethicists, and other stakeholders, diverse perspectives can be integrated to develop innovative solutions. Increased interdisciplinary training and education can foster a common understanding among stakeholders, facilitating more effective collaboration and problem-solving^22,24^.

Furthermore, innovative engineering and material science solutions are required to enhance the fidelity of micro-engineered models. These solutions should focus on accurately replicating neuronal architecture and achieving biomimicry^22^ to improve the relevance and effectiveness of micro-engineered models in BMI research and development^47^. By overcoming these challenges, the future of BMI research holds immense potential to improve the lives of individuals with neurological disorders and advance our understanding of brain-machine interactions (Table 3).

**Table 3:** Future aspects, possible trends, and challenges pertaining to each aspect with their probable solution.

Aspect	Trend	Challenge	Solution
Technology integration	Integration of AI and ML	Technical challenges	Collaborative research, innovation
Miniaturization	Smaller, implantable BMIs	Long-term stability of neural networks	Improved materials, research
Noninvasive BMIs	EEG-based systems	Data security, privacy	Ethical protocols, secure systems
Neural network replication	Microengineered Models	Biomimetic challenges	Innovative engineering solutions
Ethical considerations	Responsible BMI development	Ethical concerns	Regulatory standards, ethics committees
Interdisciplinary efforts	Collaboration across disciplines	Multifaceted challenges	Shared education, research networks

## Conclusion

MNNs hold vast potential for therapeutic applications and advancing our understanding of neural processes in BMIs. Explored in this paper, these models can revolutionize how we treat neural disorders and rehabilitate injured brain tissue, offering valuable insights into neural connectivity and signal processing. Despite challenges and ethical considerations, the undeniable revolutionary potential of BMIs, especially when combined with AI in noninvasive systems like EEG-based BMIs, promises exciting developments. Micro-physiological models present unprecedented opportunities for previously unthinkable therapies, providing hope for individuals with conditions like paralysis and neurodegenerative diseases. The revolutionary potential of BMIs cannot be overstated, and our findings underscore the need for further exploration and creative thinking in this domain. As we anticipate exciting future developments, including more flexible and user-friendly systems, it is crucial to address technical challenges, ethical dilemmas, and regulatory barriers through interdisciplinary collaboration. Combining AI with noninvasive BMIs, such as EEG-based systems, holds promise for enhancing user quality of life. Therefore, MNNs represent a groundbreaking advancement in BMI research, with the potential to usher in a new era of brain–machine interactions. This manuscript serves as a call to action for continued study, ensuring the responsible and safe development of BMIs for therapeutic applications. By maintaining a balance between innovation and ethical responsibility, we can unlock the full potential of BMIs, benefiting both the general public and individuals with neurological disorders.

## Ethical approval

Ethical approval was not required for this article.

## Consent

Informed consent was not required for this article.

## Sources of funding

We have not received any financial support for this manuscript.

## Author’s contribution

All authors have approved the final manuscript for submission.

B.K.: supervising the draft, reviewing and editing, and project administration; O.U.: conceptualization, writing – review and designing, and project administration; S.S., A.E.H., L.N., H.A.Y., K.J.I., K.S., and K.Y.: writing the first draft and revising.

## Conflicts of interest disclosure

The author declared no conflicts of interest.

## Research registration unique identifying number (UIN)

1. Name of the registry: not applicable.
2. Unique identifying number or registration ID: not applicable.
3. Hyperlink to your specific registration (must be publicly accessible and will be checked): not applicable.

## Guarantor

Abubakar Nazir, Oli Health Magazine Organization, Research and Education Kigali, Rwanda E-mail: abu07909@gmail.com ORCID ID: 0000-0002-6650-6982

## Data availability statement

Not applicable.

## Provenance and peer review

Not commissioned, externally peer reviewed.

## References

[1] Rasheed S . A review of the role of Machine Learning techniques towards Brain–Computer Interface applications. Mach Learn Knowl Extr. 2021;3:835–862.

[2] Javaid MA . Brain-computer interface. SSRN Electron J 2013;1:15–17;

[3] Rosenfeld JV Wong YT . Neurobionics and the brain–computer interface: current applications and future horizons. Med J Aust 2017;206:363–368. 28446119 10.5694/mja16.01011

[4] Ishahak M Hill J Amin Q . Modular microphysiological system for modeling of biologic barrier function. Front Bioeng Biotechnol 2020;8:2–6. 33304889 10.3389/fbioe.2020.581163 PMC7693638

[5] Bastiaens AJ . Nano- and microengineered neuronal cell networks for brain-on-chip technology. 2019. Accessed 30 January 2024. https://research.tue.nl/en/publications/nano-and-microengineered-neuronal-cell-networks-for-brain-on-chip

[6] Lebedev MA Tate AJ Hanson TL . Future developments in brain-machine interface research. Clinics (Sao Paulo) 2011;66(Suppl 1):25–32. 21779720 10.1590/S1807-59322011001300004 PMC3118434

[7] Büyükgöze S . The brain-Computer Interface. Int Conf Techn Technol Educat 2019;ICTTE 2019):133–138.

[8] R, Birbaumer N Heetderks WJ McFarland . Brain-computer interface technology: a review of the first international meeting. IEEE Trans Rehabil Eng 2000;8:164–173. 10896178 10.1109/tre.2000.847807

[9] Xiang Z Liu J Lee C . A flexible three-dimensional electrode mesh: an enabling technology for wireless brain–computer interface prostheses. Microsyst Nanoeng 2016;2:1–8. 10.1038/micronano.2016.12 PMC6444742 31057819

[10] Habibey R Striebel J Latiftikhereshki R . Microengineered 2D and 3D modular neuronal networks represent structure-function relationship. bioRxiv 2023;1:3–4. 10.1016/j.bios.2024.116518 38924816

[11] Patil PG Turner DA . The development of brain-machine interface neuroprosthetic devices. Neurotherapeutics 2008;5:137–146. 18164493 10.1016/j.nurt.2007.11.002 PMC5084136

[12] Luttge R . Nanofabricating neural networks: strategies, advances, and challenges. J Vac Sci Technol B Nanotechnol Microelectron 2022;40:020801.

[13] Yu H Zhang Q Gu M . Three-dimensional direct laser writing of biomimetic neuron structures. Opt Express 2018;26:32111. 30650677 10.1364/OE.26.032111

[14] Lei IM Jiang C Lei CL . 3D printed biomimetic cochleae and machine learning co-modelling provides clinical informatics for cochlear implant patients. Nat Commun 2021;12:1–12. 34716306 10.1038/s41467-021-26491-6 PMC8556326

[15] Severely disabled people mind-control a robotic arm via EEG [Internet]. Medgadget. 2019. Accessed 10 November 2023. https://www.medgadget.com/2019/06/severely-disabled-people-mind-control-a-robotic-arm-via-eeg.html

[16] Karayiannis NB Venetsanopoulos AN . Applications of neural networks: a case study. Artificial Neural Networks. Springer US; 1993:299–315.

[17] Vachicouras N Tarabichi O Kanumuri VV . Microstructured thin-film electrode technology enables proof of concept of scalable, soft auditory brainstem implants. Sci Transl Med 2019;11:1–2. Accessed 10 November 2023. https://pubmed.ncbi.nlm.nih.gov/31619546/ 10.1126/scitranslmed.aax9487 31619546

[18] Bologna LL Pasquale V Garofalo M . Investigating neuronal activity by SPYCODE multi-channel data analyzer. Neural Netw 2010;23:685–697. 20554151 10.1016/j.neunet.2010.05.002

[19] Andolfi A Arnaldi P Lisa DD . A micropatterned thermoplasmonic substrate for neuromodulation of in vitro neuronal networks. Acta Biomater 2023;158:281–291. 36563774 10.1016/j.actbio.2022.12.036

[20] Wang K Man K Liu J . Microphysiological systems: design, fabrication, and applications. ACS Biomater Sci Eng 2020;6:3231–3257. 33204830 10.1021/acsbiomaterials.9b01667 PMC7668566

[21] Chandrasekaran S Fifer M Bickel S . Historical perspectives, challenges, and future directions of implantable brain-computer interfaces for sensorimotor applications. Bioelectron Med 2021;7:1–2. 34548098 10.1186/s42234-021-00076-6 PMC8456563

[22] Fekete Z Zátonyi A Kaszás A . Transparent neural interfaces: challenges and solutions of microengineered multimodal implants designed to measure intact neuronal populations using high-resolution electrophysiology and microscopy simultaneously. Microsyst Nanoeng 2023;9:1–30. 37213820 10.1038/s41378-023-00519-x PMC10195795

[23] Tomba C Villard C . Brain cells and neuronal networks: encounters with controlled microenvironments. Microelectron Eng 2015;132:176–191.

[24] Aebersold MJ Dermutz H Forró C . “Brains on a chip”: towards engineered neural networks. Trends Analyt Chem 2016;78:60–69.

[25] Shih JJ Krusienski DJ Wolpaw JR . Brain-computer interfaces in medicine. Mayo Clin Proc 2012;87:268–279. 22325364 10.1016/j.mayocp.2011.12.008 PMC3497935

[26] Vidal JJ . Toward direct brain-computer communication. Annu Rev Biophys Bioeng 1973;2:157–180. 4583653 10.1146/annurev.bb.02.060173.001105

[27] Gunduz A Sanchez JC Carney PR . Mapping broadband electrocorticographic recordings to two-dimensional hand trajectories in humans. Neural Netw 2009;22:1257–1270. 19647981 10.1016/j.neunet.2009.06.036

[28] Meng J Zhang S Bekyo A . Noninvasive electroencephalogram based control of a robotic arm for reach and grasp tasks. Sci Rep 2016;6:1–15. 27966546 10.1038/srep38565 PMC5155290

[29] Broetz D Braun C Weber C . Combination of brain-computer interface training and goal-directed physical therapy in chronic stroke: a case report. Neurorehabil Neural Repair 2010;24:674–679. 20519741 10.1177/1545968310368683

[30] Ramos-Murguialday A Broetz D Rea M . Brain–machine interface in chronic stroke rehabilitation: a controlled study. Ann Neurol 2013;74:100–108. 23494615 10.1002/ana.23879 PMC3700597

[31] Gulino M Kim D Pané S . Tissue response to neural implants: the use of model systems toward New Design solutions of implantable microelectrodes. Front Neurosci 2019;13 10–11. 31333407 10.3389/fnins.2019.00689 PMC6624471

[32] Reilly CM . Brain–machine interfaces as commodities: exchanging mind for matter. Linacre Q 2020;87:387–398. 33100387 10.1177/0024363920930882 PMC7551542

[33] Smith RJ Jr Yi T Nasiri B . Implantation of VEGF‐functionalized cell‐free vascular grafts: regenerative and immunological response. FASEB J 2019;33:5089–5100. 30629890 10.1096/fj.201801856R PMC6436645

[34] Ramos-Murguialday A Curado MR Broetz D . Brain-machine interface in chronic stroke: randomized trial long-term follow-up. Neurorehabil Neural Repair 2019;33:188–198. 30722727 10.1177/1545968319827573 PMC6420378

[35] Prinsloo S Kaptchuk TJ De Ridder D . Brain–computer interface relieves chronic chemotherapy‐induced peripheral neuropathy: a randomized, double‐blind, placebo‐controlled trial. Cancer 2024;130:300–311. 37733286 10.1002/cncr.35027

[36] Struzyna LA Wolf JA Mietus CJ . Rebuilding brain circuitry with living micro-tissue engineered neural networks. Tissue Eng Part A 2015;21:2744–2756. 26414439 10.1089/ten.tea.2014.0557 PMC4652241

[37] Mikołajewska E Mikołajewski D . Ethical considerations in the use of brain-computer interfaces. Open Med (Warsz) 2013;8:720–724.

[38] Hochberg LR Serruya MD Friehs GM . Neuronal ensemble control of prosthetic devices by a human with tetraplegia. Nature 2006;442:164–171. 16838014 10.1038/nature04970

[39] Deo DR Willett FR Avansino DT . Brain control of bimanual movement enabled by recurrent neural networks. Sci Rep 2024;14:1598. 38238386 10.1038/s41598-024-51617-3 PMC10796685

[40] Yanagisawa T Fukuma R Seymour B . MEG–BMI to control phantom limb pain. Neurol Med Chir (Tokyo) 2018;58:327–333. 29998936 10.2176/nmc.st.2018-0099 PMC6092605

[41] Kawase T Sakurada T Koike Y . A hybrid BMI-based exoskeleton for paresis: EMG control for assisting arm movements. J Neural Eng 2017;14:016015. 28068293 10.1088/1741-2552/aa525f

[42] Fiani B Reardon T Ayres B . An examination of prospective uses and future directions of neuralink: the brain-machine interface. Cureus 2021;13:3. 10.7759/cureus.14192 PMC8083990 33936901

[43] Quek S Lee T-S Goh SJA . A pilot randomized controlled trial using EEG-based brain–computer interface training for a Chinese-speaking group of healthy elderly. Clin Interv Aging 2015;10:217. 25624754 10.2147/CIA.S73955 PMC4296917

[44] Shane S Metz C Wakabayashi D . How a pentagon contract became an identity crisis for Google. The New York times 2018;A:1–3.

[45] Maynard A . How risky are the World Economic Forum’s top 10 emerging technologies for 2016? The Conversation 2016;1:1–2.

[46] Mridha MF Das SC Kabir MM . Brain-computer interface: advancement and challenges. Sensors (Basel) 2021;21:5746. 34502636 10.3390/s21175746 PMC8433803

[47] Kantawala B Ramadan N Hassan Y . Physical activity intervention for the prevention of neurological diseases. Health Sci Rep 2023;6:4–5. 10.1002/hsr2.1524 PMC10442603 37614284

[48] Manukian M Bahdasariants S Yakovenko S . Artificial physics engine for real-time inverse dynamics of arm and hand movement. PLoS One 2023;18:e0295750. 38091328 10.1371/journal.pone.0295750 PMC10718432

[49] Musk E Neuralink . An integrated brain-machine interface platform with thousands of channels. J Med Internet Res 2019;21:e16194. 31642810 10.2196/16194 PMC6914248

[50] Simon C Bolton DAE Kennedy NC . Challenges and opportunities for the future of brain-computer interface in neurorehabilitation. Front Neurosci 2021;15:2–4. 10.3389/fnins.2021.699428 PMC8282929 34276299

